# Precise Amplitude and Phase Determination Using Resampling Algorithms for Calibrating Sampled Value Instruments

**DOI:** 10.3390/s20247345

**Published:** 2020-12-21

**Authors:** Yeying Chen, Enrico Mohns, Michael Seckelmann, Soeren de Rose

**Affiliations:** Instrument Transformers and Sensors, Electrical Energy Measuring Techniques, Physikalisch-Technische Bundesanstalt (PTB), 38116 Braunschweig, Germany; Enrico.Mohns@ptb.de (E.M.); Michael.Seckelmann@PTB.DE (M.S.); soeren.de-rose@ptb.de (S.d.R.)

**Keywords:** resampling algorithm, sampled values, SV-based instrumentation, SV-based calibration, phase synchronisation, sinc interpolation

## Abstract

Sampling-based calibration systems for calibrating “Sampled Value” (SV)-based instruments for substation automation require synchronised and time-aligned sampling processes. As the signal frequency of the power grid is always asynchronous to the standardised sampling frequencies according to IEC 61869-9, the sampled waveforms of the calibration system and of the SV-based device under test can be resampled to be synchronised and to allow better accuracy in the following measurements based on the Discrete Fourier Transform (DFT) of the resampled waveforms. The paper presents simulations and results for different resampling algorithms. A modified sinc interpolation method with a finite impulse response (FIR) is presented. The deviation of the results for the root mean square (RMS) and phase angle is in the order of 10^−8^ *V*/*V* (or rad) for normalised frequencies of up to 20% of the sampling frequency. No practical degradation in the presence of noise and harmonics could be observed. In addition, laboratory experiments demonstrate the realization of the proposed resampling process in the future SV-based calibration systems for SV-based instrumentation.

## 1. Introduction

**Figure 1:**
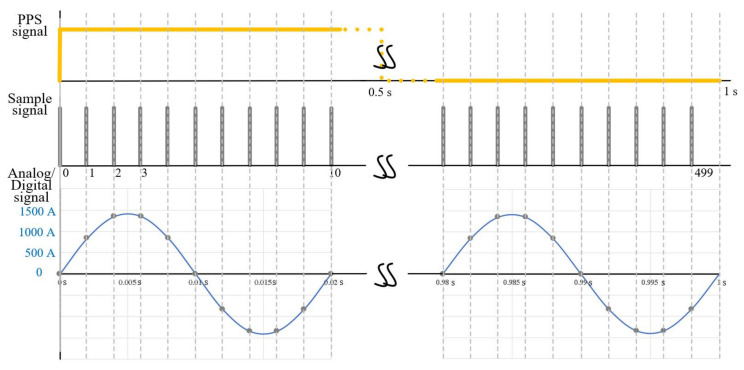
Principle scheme of the sampling process for the SV-based devices according to standard IEC 61869-9. For illustration purpose, a non-standardized sample rate of 500 S/s (10 samples per period at 50 Hz) is applied.

In the field of electrical alternating current (AC) power and energy measurements, several modern measuring systems rely on internal sampling processes from which voltages and currents, their phase angles or their spectra are to be determined. Moreover, some instruments, such as phasor measurement units, are synchronised to a common pulse-per-second (PPS) clock, distributed, e.g., via global navigation satellites, to allow the absolute phase with respect to this clock to be determined [1]. In the European funded metrological research project 17IND06 “FutureGrid II” [2], the research on and realisation of metrological calibration systems for the digital instrumentation in high-voltage substations will be carried out. In such substations, traditional instrument transformers and associated analogue metering and protection devices will be replaced or retrofitted with sampled value (SV)-based [3] digital instrumentation. One important device in such a measurement chain is the merging unit (MU)/the stand-alone merging unit (SAMU) [4] which performs the analogue-to-digital (AD) sampling process of up to four voltage instrument transformers and of up to four current instrument transformers. The SAMU also performs the phase synchronisation between all eight channels by ensuring simultaneous sampling of the internal AD converters, as well as the alignment and time-stamping of the SV to the PPS clock. Typical standardised sampling rates are between 4 kS/s and 15.36 kS/s for AC applications [3]. Figure 1 demonstrates the sampling process based on the standard IEC 61869-9. From a model perspective, it is important to understand that the sampling process, which can be considered mathematically as the generation of an ordered sequence of samples of a continuous signal x(t), at regular, specific instants of time. The amplitude quantisation according to the standard IEC 61869-9 is 10 mV for the voltage sequences and 1 mA for the current sequences.

According to the standard IEC 61869-9, the standard sample rates for the SV-based instruments are presented in Table 1.

A calibration system for such a SAMU must not only provide suitable reference values of voltage and current (e.g., root mean square (RMS) values or the harmonic content of the signals). It must also provide a synchronisation and alignment to the PPS in order to allow calibrating the phase angle between the SAMU voltage and current channels and to further allow calibrating the absolute phase angle of each channel with respect to the PPS. Synchronisation for an accurate determination of the power quality parameters can be achieved by various methods regarding to the different aspects of the calibration system. For instance, in a laboratory environment, the synchronisation of the sampling processes of the reference measuring system is already possible by synchronising the sampling frequency *f*_S_ to the fundamental frequency *f*_1_ of the signal and by starting the sampling process of the reference system with the PPS [1,5,6]. Moreover, the software-based algorithms in the frequency domain have been used to synchronise an asynchronously running sampling to the measured signal for the measuring system [7,8]. Besides this, algorithms related to the Discrete Fourier Transform (DFT) analysis techniques [9,10,11] have been applied for the synchronisation. Furthermore, when the frequency of the digitised signal is not as expected as the frequency based on the sampling clock of the measurement system, a method has been used that relies on a time domain interpolation technique to modify the sampling rate of the captured signal in software [12]. Even in other metrological applications such as ac power standards, at least the synchronisation of *f*_S_ to *f*_1_ can easily be attained in the laboratory environment by the hardware synchronisation of an analogue to digital converter (ADC) internal master clock (e.g., 10 MHz) with the master clock of a signal generator [13,14,15] or of an analogue or digital phase-locked loop (PLL) circuits which provide a synchronised sampling frequency *f*_S_ which is an integer multiple of the fundamental frequency *f*_1_ [8,16,17]. To achieve the synchronisation to the signal frequency and to the PPS, a modified sinc interpolation in the time domain, proposed in this paper, performs a resampling process, where the length of the new dataset is aligned to a maximum possible integer number of the signal period. Therefore, no further windowing is required for the subsequent DFT analysis [18]. Additionally, a phase correction is described, which is required to preserve phase accuracy to the PPS. An advantage is that the resampling based analysis is not only accurate for the power frequency (i.e., the fundamental) but also for the wideband spectral content of the signal.

In this paper, different resampling algorithms for choosing the most suitable algorithm and implementing it in a new calibration system for digital current transformers [19] have been simulated. Resampling algorithms using polynomial interpolations based on a quadratic function and a cubic function are presented in this work for comparison to the resampling algorithm using the modified sinc interpolation [20,21,22]. The attainable accuracy after applying a DFT to a resampled series of sampled values in terms of the RMS error and the error of the absolute phase angle for the fundamental as well as the simulated harmonics is determined. LabView-based software has been developed to conveniently perform the simulations as well as the calculations and statistics and the data transfer. The presented algorithms strongly rely on the precise determination of the fundamental frequency *f*_1_ of the (original) series of sampled values. In the presented algorithms, we make use of a LabView functionality for extracting the frequency based on the IEEE-STD-1057 four-parameter sine wave fit algorithm. It is not the intention of the authors to incorporate an extensive mathematical analysis, instead results for each test case are given. For the evaluation of the resampling algorithms, other error sources, typically associated with ADCs such as quantisation errors, or device internal phase displacements such as from the analogue input filtering or the ADC’s internal digital filter are not considered (see Section 3.1). This allows us to exclusively assess the attained accuracy of the resampling algorithm. However, quantisation errors which are due to the number resolution of the SV communication protocol are integrated into the simulation by using a self-developed SV-based generator and receiver (see Section 3.2).

## 2. Designs and Methods

The designs and methods are divided into two parts: the designs of the resampling algorithms and the application methods for the practical validation of the simulated resampling algorithms.

### 2.1. Resampling Process and Simulated Algorithms

**Figure 2:**
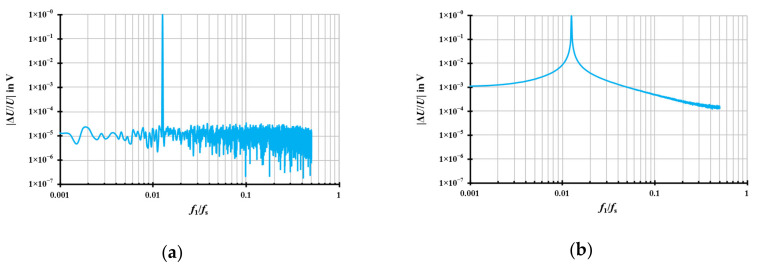
Discrete Fourier Transform (DFT) spectra of the synchronous signal; (**b**) The DFT spectra of the asynchronous signal. The signals are given for *f*_S_ = 4000 Hz, *T*_W_ = 1 s, *U*_1_ = 1 V, *f*_1_ = 50.1 Hz, *φ*_1_ = 0 ° and *u*_Noise_ = 1 mV without harmonics.

For the resampling simulation, the generation of a time-discrete waveform (i.e., a sampled signal) is proposed, which covers three components: a fundamental sinusoidal signal, a noise signal (white noise) and a series of sinusoidal signlas to simulate several harmonics. The digitised signal, as a signal sequence *u*[*k*], is given by:

(1) $$u[k]\;=\;\sqrt{2}\cdot U_{1}\cdot \sin\left(2\pi \cdot f_{1}\cdot k\cdot T_{S}+\varphi_{1}\right)+u_{\mathrm{Noise}}+\sum_{m>1}\sqrt{2}\cdot U_{m}\cdot \sin\left(2\pi \cdot f_{m}\cdot k\cdot T_{S}+\varphi_{m}\right),$$

where *k* = 0, 1, 2, …, *N* − 1, *k* refers to the original sampling value index of the signal sequence, *N* refers to the original sample number of the signal sequence and *T*_S_ is the sampling time of the sampling process. *U*_1_, *f*_1_, and *φ*_1_ refer to the RMS voltage, the frequency and the phase angle of the fundamental sinus waveform signal, respectively; *u*_Noise_ refers to the RMS value of the noise signal that is generated by a white noise random generator with a rectangular distribution, the variable *m* is an integer which refers to the order of the harmonic series. *U*_m_, *f*_m_, and *φ*_m_ refer, respectively, to the RMS voltage, the frequency and the phase angle of the *m*^th^ harmonic sinusoidal. The generated sampling values of the signal sequence, which are mathematically computed from Equation (1), are regarded as the reference sampling values for the resampling process. The Discrete Fourier Transform (DFT) spectra of a signal which is synchronised (a) and not synchronised (b, asynchronous signal) to the sampling frequency are shown in Figure 2.

Theoretically, the sampling values of the sinus waveform signal sequence *u*[*k*] are sampled by a sampling period *T*_S_ (i.e., the sampling rate *f*_S_ = 1/*T*_S_) in a window of the length *T*_W_. A waveform with 1 V RMS, 4000 samples/s and a window of 1 s has been generated with a fundamental frequency *f*_1_ that is not synchronous to the sampling process. The corresponding asynchronous sampling results in the frequency domain are displayed in Figure 2b as the DFT spectrum showing the RMS-scaled amplitude behaviour. It is obvious that the leakage of the spectrum will lead to inaccurate results for the RMS values as well as for the phase angle (not shown). The idea of the proposed resampling is that the sampling values of the sinus waveform signal sequence *u*[*k*] are resampled at a (new) resampling rate *f*_S_’ (i.e., the resampling period *T*_S_’ = 1/*f*_S_’) and that a new window of the length *T*_W_’ is calculated which contains an integer multiple of the measured signal period *T*_1_ = 1/*f*_1_. The synchronised DFT results in the frequency domain are illustrated in Figure 2a showing the amplitude at *f*_1_ only and the noise floor from the generated noise signal.

#### 2.1.1. Program structure of the Resampling Process

**Figure 3:**
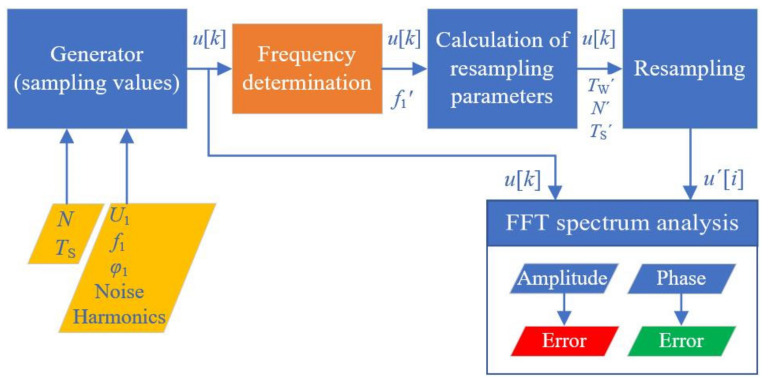
Scheme of the resampling process.

The program structure of the resampling process, introduced in Figure 3, is mainly made up of five function blocks: the sampling value generator, the frequency determination of the sampling values, the calculation of the resampling parameters, the resampling and the Fast Fourier Transform (FFT) spectrum analysis. The resampling block contains three different resampling algorithms for investigation which are based on a quadratic interpolation function, on a cubic interpolation function and a modified sinc interpolation.

First of all, a set of discrete signal samples *u*[*k*] is generated for the simulation according to Equation (1). The generation is realised with the appropriate setup of the sampling parameters (*N*, *T*_S_) and the characteristic signal parameters (*U*_1_, *f*_1_, *φ*_1_, the noise parameter and the additional harmonic parameters). As a result, the sequence of the generated discrete values is sent by the generator. After the digital transmission, the sequence is received for resampling. As the resampling process must be executed by using recognised sampling parameters of the original sequence *u*[*k*], such as *T*_S_, *N* and the detected signal frequency, the received sequence *u*[*k*] needs to be processed in order to extract these required parameters for the resampling. This is done in the two function blocks between the generator and the resampling, shown in Figure 3, which analyse the discrete sampling samples from the generator. The first block (labelled as “Frequency determination” in Figure 3) determines the fundamental frequency *f*_1′_ by using the IEEE-STD-1057 four-parameter sine wave fit algorithm [23] based on the received original sampling sequence *u*[*k*]. After that, in the second block (labelled as “Calculation of resampling parameters” in Figure 3), the required parameters for the resampling process are calculated, which refer to the total time window *T*_W_’, the number of the samples *N*’ and the sampling time *T*_s_’. The subsequent calculation of the resampling sequence *u*’[*i*] is performed in the block (labelled as “Resampling” in Figure 3).

The selection of the different resampling algorithms subsequently leads to a different number of the samples *w* that must be at least cut off at the beginning and at the end of the original sampling sequence for the resampling process. This number is set at *w* = 2 for the quadratic interpolation, *w* = 3 for the cubic interpolation and *w* = *N*_F_ for the modified sinc interpolation. An optional time delay *T*_Del_ at the beginning of the original sampling sequence is determined by the user. The new resampling parameters can be obtained through:

*T*_W_’ = *T*_W_ − (*w* · *T*_s_ + *T*_Del_), (2)

*N*’ = 2*^Ceil^*^(*Log*(*N*;2))^, (3)

*T*_s_’ *=* (*N*_per_’/*T*_1_)/*N*’, (4)

where the number of the resampling period *N*_per_’ of the fundamental signal is calculated by *N*_per_’ = *Floor*(*T*_W_’/*T*_1_). Further detailed operations of the parameters are presented in Section 2.1.2 and Section 2.1.3. The number of samples *N*’ was chosen to be a binary number, for which the new sampling rate *f*_S_´ = 1/*T*_S_´ is higher than the original sampling rate.

Eventually, the FFT spectrum analysis of the resampling values *u*’[*i*], where *i* refers to the resampling value index of the signal sequence, is used for the determination of *U*_DFT_(*f*), which refers to the complex signal parameters of the resampled signal sequence. These results are used for the comparison to the known signal parameters of the generated original signal sequence. The quality of the simulated algorithms is evaluated in terms of the (positive; the use of the positive errors allows logarithmic diagrams to be used) relative amplitude errors |Δ*U*/*U*| and the (positive) phase errors |Δ*φ*| that are determined by |Δ*U*/*U*| = |[|*U*_DFT_(*f*)| − *U*_1_]/*U*_1_| for the amplitude evaluation and |Δ*φ*| = |(arg{*U*_DFT_(*f*)} − *φ*_1_| for the phase evaluation.

#### 2.1.2. Interpolations Based on a Quadratic and a Cubic Function

The interpolations based on a quadratic and a cubic function principally both match the polynomial interpolation which is applied to find the coefficient of a polynomial function based on given data through the corresponding quadratic and cubic functions. The detailed mathematical analysis of the resampling algorithms based on a quadratic interpolation and a cubic interpolation is presented in Appendix A. Additionally, the detailed determination of the first resampling sample for the sake of a phase correction is explained in Section 2.1.4.

#### 2.1.3. Interpolation Based on a Modified Sinc Function

The interpolations based on a quadratic and a cubic function, introduced in Appendix A, are considered as a simple and basic method for the resampling process. However, the polynomial interpolation is limited by the polynomial degrees. Higher degrees may contribute to more accurate resampling results, but higher degrees can also lead to the undesirable properties in the resampled waveform. Therefore, an interpolation based on a modified FIR-approximated sinc function has been chosen as the interpolation kernel, which in theory [24] can be used in resampling processes where the resampled waveform contains the same information as the original waveform. Thus, calculations of the spectral composition using a DFT lead to exact numerical results.

Generally, a sinc function [25] is expressed as sinc(*x*) = sin(*x*)/*x*. Interpolation based on a sinc function (i.e., the Whittaker–Shannon interpolation formula [26]) is supposed to reconstruct a continuous-time bandlimited function *x*(*t*) from a given signal sequence *x*[*k*]. The sinc function for the resampling interpolation denotes the normalised sinc function, expressed as sinc(*x*) = sin(π*x*)/π*x*. The generalised sinc interpolation equation is denoted by:

(5) $$x(t)=\sum_{k=-\infty }^{+\infty }x[k]\cdot \mathrm{sinc}\left(\frac{t}{T_{s}}-k\right).$$

The generalised sinc interpolation equation consists of parameters of the signal sequence and parameters of the normalised sinc function. When the *x*[*k*] sequence is bandwidth-limited to less than the Nyquist frequency, *x*(*t*) is a perfect reconstruction of the original function based on the sampling theorem. Combined with resampling parameters, *t*/*T*_s_ can be replaced through *i* · *T*_s_’/*T*_s_. The sampling samples of the given signal sequence, *x*[*k*] = *u*[*k*], are generated according to Equation (1). The resampling value is calculated through the interpolation based on a modified sinc function:

(6) $$u^{\prime }\left(i\right)=\sum_{k=0}^{N-1}u\left[k\right]\cdot \mathrm{Intp}\left(x\right),$$

where *x* = *i* · *T*_s_’/*T*_s_ − *k*. The modified interpolation kernel is expressed as follows:

(7) $$\mathrm{Intp}\left(x\right)=\left\{\begin{array}{c} A\cdot \cos^{q}\left[\frac{\pi }{N_{F}}\cdot x\right]\cdot \mathrm{sinc}\left(\pi \cdot x\right),\;-\frac{N_{F}}{2}<x<+\frac{N_{F}}{2}\\ 0,\;\mathrm{otherwise} \end{array}\right..$$

**Figure 4:**
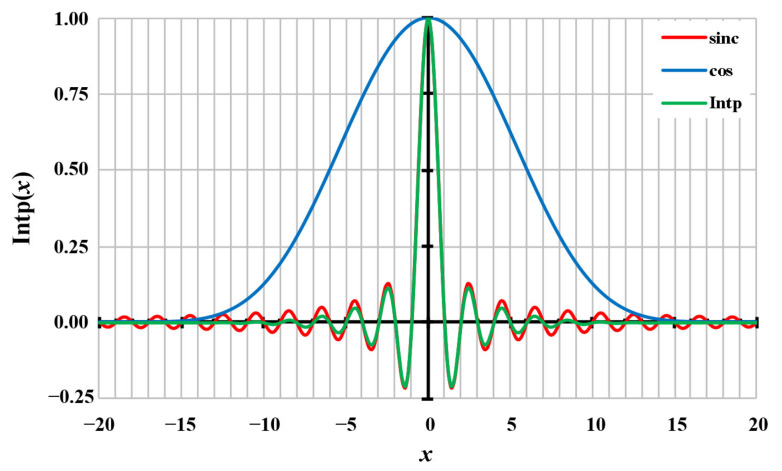
The composition of the interpolation kernel Intp(x) (green curve) can be seen by the pure sync function (red) and the modifying cos^q^ function (blue curve) for *N*_F_ = 40 and *q* = 6.

According to Equation (7), the sinc function for the resampling process is limited to the maximum window size *N*_F_. The truncation is achieved with windowing using a cos function with additional exponents *q*. The value *A*, which is usually one, is used for scaling, if required. The detailed composition of the modified interpolation kernel is illustrated in Figure 4. The detailed determination of the first resampling sample is explained in Section 2.1.4.

The use of the window size *N*_F_ optimises the algorithm, so that only the number of samples in the considered period has to be taken into account. This drastically reduces the number of multiplications, sine and cosine calculations.

#### 2.1.4. Phase Synchronisation

The synchronisation based on Equation (2) considers a user-defined time delay *T*_Del_. The time instant *t*_0_’of the first resampled value is chosen to be equal to the time instant *t*_i_ for which *t*_i_ ≥ *T*_Del_. In the case of the sinc interpolation method used, the first possible resampled value is located at *t*_0_’ = (*N*_F_/2 + 1) · *T*_s_. To synchronise the phase of the resampling samples, a phase correction is applied and is based on the time instant *t*_0_’ of the first sample *u*’(0) of the resampling process. The correction is done by calculating 2·π·*f*·*t*_0_’, where *f* is the corresponding frequency of interest.

### 2.2. Emulation Scheme of the SV-Based Communication

**Figure 5:**
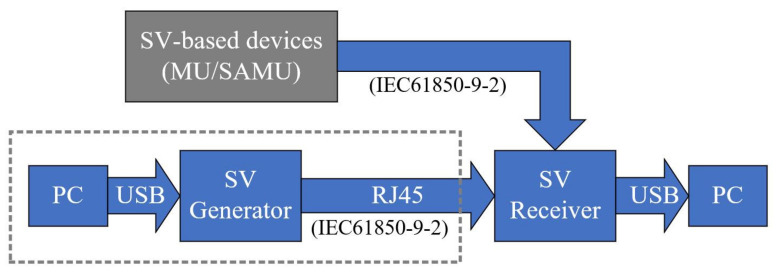
Emulation scheme of the SV-based communication.

The emulation scheme, introduced in Figure 5, realise the laboratory implementation of the SV-based communication. A computer (PC) is not guaranteed to receive all the digital data based on the standard IEC 61850-9-2 [27] without problem. Therefore, a microcontroller-based SV receiver box and the corresponding LabView-based program on the PC are especially designed for receiving sampled values from the digital instruments using IEC 61850-9-2 communication protocols. To avoid any loss of sampled values, the SV receiver box offers a time buffer for the digital data processing. In praxis, the sampled values are sent from the SV-based devices, e.g., SAMU/MU (shown as the grey block in Figure 5). For laboratory validation experiments, a microcontroller-based SV generator box and the corresponding LabView-based program on the PC (the blocks in the grey dotted rectangle in Figure 5) are designed as a substitute for the SV-based devices with a 1 mA current resolution and 10 mV voltage resolution. In this case, the signals for the subsequently arranged evaluations from the SV-based devices (e.g., SAMU or digital energy meter) can be generated much simpler and adjusted for any special research requirements.

The method used for this paper is mainly made up of four parts: two LabView-based software programs on the PC blocks and two microcontroller-based hardware blocks (shown as the blue rectangle blocks in Figure 5).

Firstly, the LabView-based software platform on the PC (the left PC block in Figure 5) program the microcontroller of the SV generator box. The software for the SV generator box defines the sampling parameters (*N*, *T*_S_) and the characteristic signal parameters (*U*_1_, *f*_1_, *φ*_1_, the noise parameter and the possible harmonic parameters), which are introduced in Section 2.1.1. Secondly, the programmed SV generator box generates and sends the user-defined sampled values according to the standard IEC 61850-9-2 to the SV receiver box. Thirdly, the SV receiver box receives the sampled values with the user-defined time buffer that is programmed by the LabView-based software platform on the PC (the right PC block in Figure 5). Then, the sampled values are transmitted without any loss from the SV receiver box to the PC. Finally, the received sampled values can be used for the further computations (e.g., for synchronisation by using the designed resampling algorithms).

## 3. Results and Validation of the Resampling Process

The results and validation are presented through two parts: the simulation results without consideration of any quantization errors due to the standardized SV communication protocol and the results by using the self-developed microcontroller-based SV devices, which are introducing the quantization errors to the sampled values. The resampling process is simulated under a LabView environment.

### 3.1. Simulation Results

The simulations with the three resampling algorithms are divided into three parts: the general simulations, the simulations of the frequency response and the simulations with the harmonic interactions. The simulation results were firstly saved from the LabView program as the dataset, then analysed and presented in the diagrams. Before the simulations among the three resampling algorithms, the parameters of the interpolation based on a modified sinc function were investigated for a proper setup. As a result, the resampling algorithm of the modified sinc interpolation was set with *q* = 6 and *N*_F_ = 40 as a standard setup for the rest of the simulations. The simulations related to the noise influence were investigated with the RMS noise levels *U*_Noise_ of 1 μV, 10 μV, 100 μV and 1 mV. The results showed that there was no unusual behaviour depending on the different noise levels. With the chosen sampling parameters of *f*_S_ = 4 kHz and the window *T*_W_ = 1s (frequency resolution *f*_res_ = 1/*T*_W_), the noise floor in the spectra agrees with the theoretical level of *U*_Noise_/√(*f*_S_/2) · √(*f*_res_). For a noise level of 1 mV, the noise floor is about 20 µV and is evenly distributed across the frequency range up to *f*_S_/2.

#### 3.1.1. General Investigation Simulations

General simulations with the three resampling algorithms were executed with a pure sinusoidal waveform without the noise and the harmonic series. In this case, the qualities of the different resampling algorithms were evaluated without any disturbances. The two essential indicators of the algorithm qualities are the relative amplitude errors |Δ*U*/*U*| between the sampling sequence and the resampling sequence and the computation time for the calculation of the whole resampling sequence.

**Figure 6:**
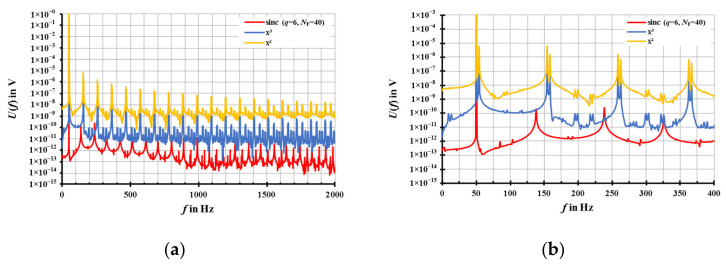
(**a**) Full DFT spectra of the resampled signals by using the quadratic interpolation (orange), the cubic interpolation (blue) and the modified sinc interpolation (red). (**b**) Detailed DFT spectra of the resampled signals to see the fragments of each interpolation (quadratic, cubic, sinc). The signals are simulated with parameters of *f*_S_ = 4000 Hz, *T*_W_ = 1 s, *U*_1_ = 1 V, *f*_1_ = 50.1 Hz, and *φ*_1_ = 0° without any noise or harmonics.

Firstly, the resampling results of the different resampling algorithms, shown in Figure 6 with the RMS-scaled amplitude *U*_DFT_(*f*), were simulated with the corresponding standard setups labelled in Figure 6. It is obvious to see from Figure 6a that the resampling results of the modified sinc interpolation are better than the interpolation based on the cubic function. The results of the interpolation based on the cubic function are better than the interpolation based on the quadratic function. All algorithms show no leakage, but a computational noise floor and fragments, which are shown in more detail in Figure 6b. However, the values of the fragments are small enough so that the main results will not be affected.

As a result, the computation time for the calculation of the whole resampling sequence of each resampling algorithm was recorded together with the simulation results in Figure 6. Compared to the fastest algorithm, the interpolation based on the quadratic function (around 1 ms), the interpolation based on the cubic function is only slightly slower. The modified sinc algorithm is on average, 30 times slower (30 ms) than the quadratic algorithm. At a sampling rate of 20 kS/s and a window time of 1 s, the computation time is about 8 ms for the quadratic interpolation, 9 ms (cubic) and 250 ms (sinc).

Nevertheless, the amplitude errors of the modified sinc algorithm are below 10^−9^ *V*/*V*, while the quadratic and the cubic algorithms exhibit errors in the order of 5·10^−7^ *V*/*V*. The phase errors (not shown in Figure 6) of the cubic and sinc interpolation algorithms are well below 10^−9^ rad, while for the quadratic interpolation, a systematic phase error of about 20 µrad has been observed.

Additionally, signal sequences with different initial phases *φ*_1_ between ±180 ° were simulated for the resampling process. As a result, the errors of the quadratic algorithm are below 10^−6^ for the amplitude errors and in the order of 2·10^−5^ for the phase errors. The errors of the cubic algorithm and sinc algorithm are below 10^−9^ for the amplitude errors and below 10^−9^ for the phase errors.

Further simulations (repetitions up to 1000) with fluctuating (random) parameters of the generated signal sequence (*U*_1_, *φ*_1_) were executed. Here, *U*_1_ ranges from 0.95 to 1.05 V, and *φ*_1_ is in the range ±180 °. These results showed that the fluctuating parameters have no significant effect on the accuracy of the resampling algorithms, which means that the computational accuracy of each algorithm does not depend on smaller fluctuations around the “rated” values of the parameters used.

#### 3.1.2. Frequency Response

**Figure 7:**
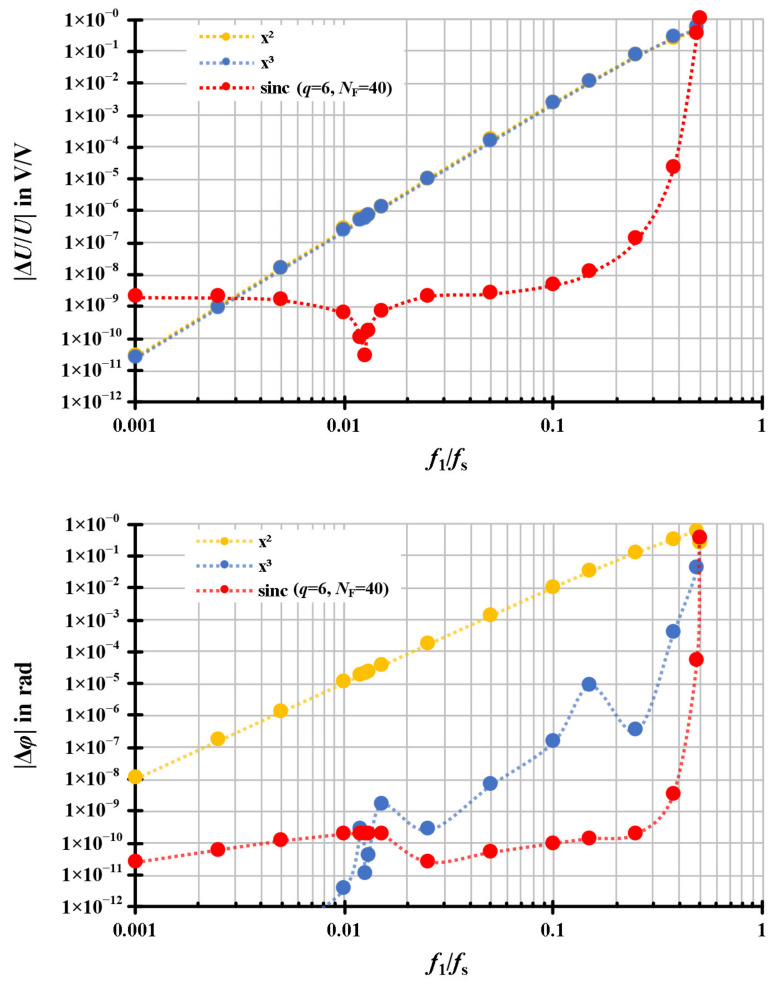
Frequency responses of the quadratic, the cubic and the modified sinc interpolations. The signals are simulated with parameters of *f*_S_ = 4000 Hz, *T*_W_ = 1 s, *U*_1_ = 1 V, and *φ*_1_ = 0° without noise and the harmonic series. The orange dots represent the results of the interpolation based on the quadratic function. The blue dots represent the results of the interpolation based on the cubic function. The red dots represent the results of the modified sinc interpolation.

The simulations of the frequency response concentrate on the algorithm behaviours associated with the various signal frequencies *f*_1_. The other parameters of the sampling signal sequence, except *f*_1_, were set the same as the signal sequence for simulations in Section 3.1.1, generated with a pure sinusoidal waveform without noise and the harmonic series. Based on the sampling rate *f*_S_ = 4000 Hz, the resampling process was repeated for each algorithm with different signal frequencies *f*_1_ from 4 Hz to almost 2 kHz, which is the Nyquist frequency. The simulation results of the different resampling algorithms are exhibited in Figure 7 with the frequency responses of the relative amplitude errors |Δ*U*/*U*| (the diagram above in Figure 7) and of the absolute phase errors |Δ*φ*| (the diagram below in Figure 7). The x-axis of the diagrams is normalised by the ratio of the signal frequency and the resampling frequency *f*_1_/*f*_S_, which is 0 < *f*_1_/*f*_S_ < 0.5. The absolute phase errors |Δ*φ*|, presented in Figure 7, are synchronised to the sampling signal and corrected with a 90 degree phase displacement owing to the FFT analysis, which extracts the phase angle related to a cosine instead of a sine.

According to Figure 7, it is apparent that the relative amplitude errors |Δ*U*/*U*| and the absolute phase errors |Δ*φ*| both have rising trends with increasing frequencies. The ratio *f*_1_/*f*_S_, ranges from 0.001 to 0.5 and complies with the simulated signal frequency *f*_1_, ranging from 4 Hz to 2 kHz.

For the relative amplitude errors, the results of the resampling algorithms based on the quadratic and the cubic interpolations, shown as the orange dots and the blue dots in Figure 7 (above), have almost identical frequency responses with the errors of below 10^−10^ *V*/*V* at 4 Hz. Besides this, the relative amplitude errors are around 0.2 *V*/*V* up to a frequency up to 40% of the sampling rate. The relative amplitude errors are increasing with the ratio *f*_1_/*f*_S_, i.e., the signal frequency *f*_1_. In contrast, the relative amplitude errors of the modified sinc algorithm, shown as the red dots in Figure 7 (above), are stably in the range of 10^−9^ *V*/*V* to 10^−6^ *V*/*V* with a frequency up to 40% of the sampling rate. With higher frequencies (>0.4 · *f*_S_), even the modified sinc algorithm is no longer accurate for the resampling process.

For the absolute phase errors, the results of the resampling algorithms based on the quadratic interpolations, shown as the orange dots in Figure 7 (below), are below 10^−5^ rad with a frequency of up to 50 Hz (80 samples per period or *f*/*f*_S_ = 0.0125). The absolute phase errors of the resampling algorithms based on the cubic interpolations, shown as the blue dots in Figure 7 (below), are below 10^−5^ rad with a frequency up to 1 kHz (four samples per period or *f*/*f*_S_ = 0.25). The absolute phase errors are increasing with the ratio *f*_1_/*f*_S_, i.e., the signal frequency *f*_1_. In contrast, the absolute phase errors of the modified sinc algorithm, shown as the red dots in Figure 7 (below), are stably in the range from 10^−9^ rad to 10^−6^ rad with a frequency of up to 40% of the sampling rate.

#### 3.1.3. Harmonics Interactions

The simulations with the harmonic interactions, which are one of the representative indicators related to the power quality, focus on investigating the algorithm quality under various harmonic interactions. The simulations contain the investigation on the fundamental sinus waveform signal with a single harmonic interaction, the investigation on the fundamental sinus signal with a harmonic series and the investigation on the fundamental sinus signal with a square waveform.

**Figure 8:**
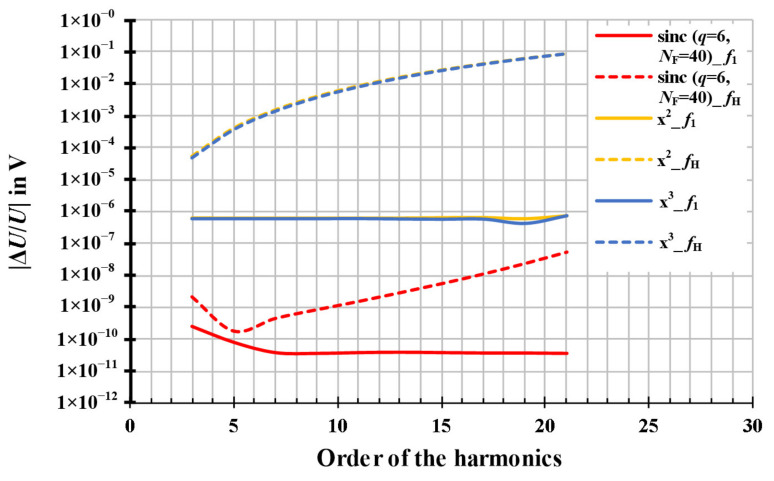

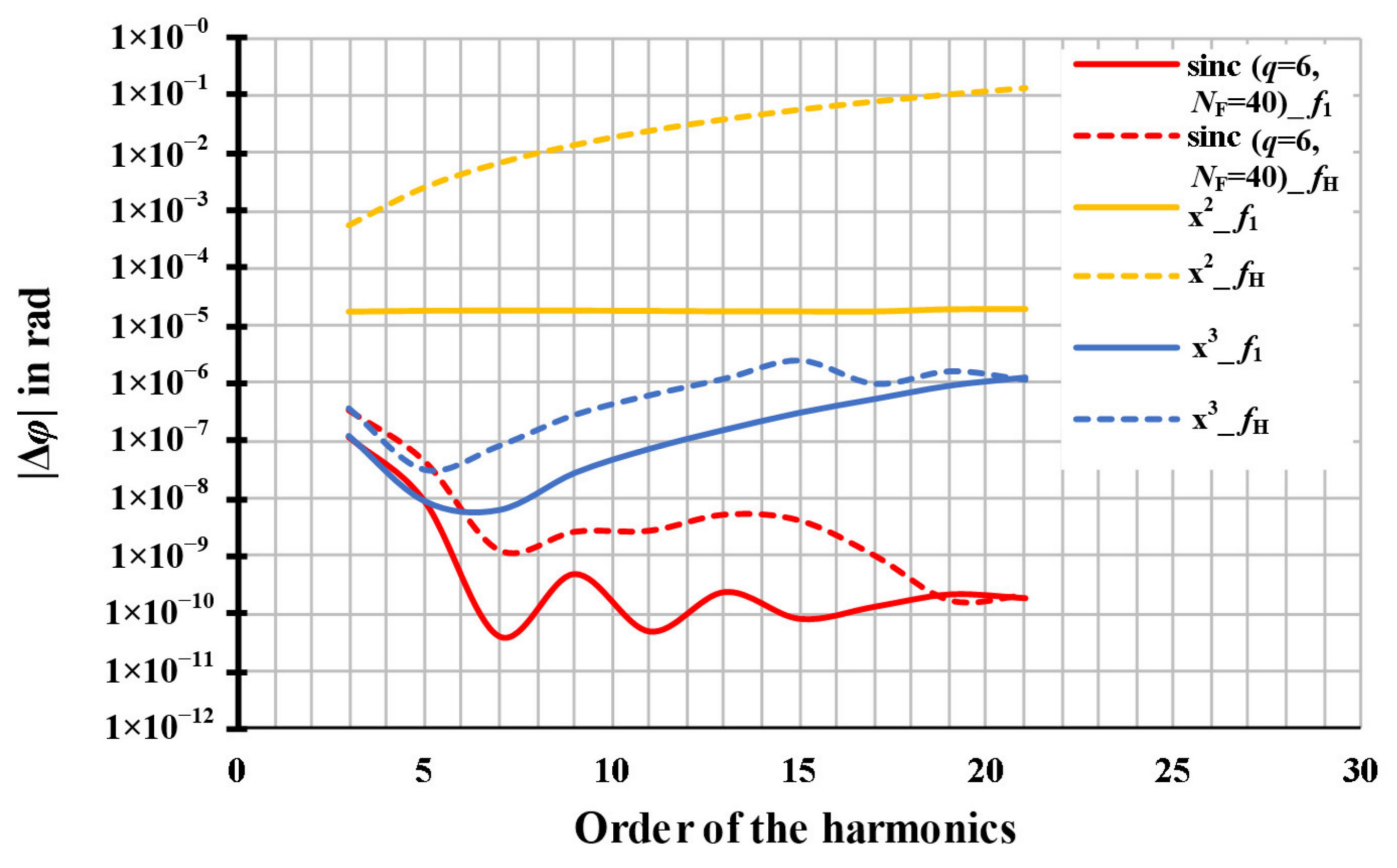
The resampling amplitude and phase error of the fundamental sinus waveform signal with a single harmonic. The signals are simulated with parameters of *f*_S_ = 4000 Hz, *T*_W_ = 1 s, *U*_1_ = 1 V, *f*_1_ = 50.1 Hz, *φ*_1_ = 0°, *U*_H_ = 0.1*U*_1_, *f*_H_ = (2*h* + 1)*·f*_1_, and *φ*_H_ = 0°, where *h* is an integer and *h* = 0, 1, 2, …, 10, without noise signal. The orange curves represent the results of the interpolation based on the quadratic function. The blue curves represent the results of the interpolation based on the cubic function. The red curves represent the results of the modified sinc interpolation.

Firstly, the simulation results of the fundamental sinus waveform signal with a swept single harmonic are illustrated in Figure 8. The order of the simulated harmonic signal is 2*h* + 1, where *h* is the positive integer number. The amplitude of the simulated harmonic signal is 10% of the amplitude of the fundamental signal.

For the relative amplitude errors, the results of the resampling algorithms based on the quadratic and the cubic interpolations, shown as the orange curves and the blue curves, respectively, in Figure 8 (above), have almost the same errors for both the fundamental signal and the harmonic signal. The relative amplitude errors of the fundamental signal are flat below 10^−6^ *V*/*V* with the order of the harmonic signal from 3 to 21. The relative amplitude errors of the harmonic signal are increased from about 10^−4^ *V*/*V* with the order of the harmonic signal to 10^−1^ *V*/*V*. The relative amplitude errors of the modified sinc algorithm, shown as the red curves in Figure 8 (above), are similar to the quadratic and the cubic algorithms. The relative amplitude errors of the fundamental signal are flat below 10^−9^ *V*/*V* with the order of the harmonic signal from 3 to 21, while the relative amplitude errors of the harmonic signal are below 10^−7^ *V*/*V*.

For the absolute phase errors, except the resampling algorithms based on the quadratic function, the results of the resampling algorithms based on the cubic function and the modified sinc interpolation, shown as the blue curves and the red curves, respectively, in Figure 8 (below), are below 10^−5^ rad with the order of the harmonic signal from 3 to 21.

Furthermore, the resampling of the fundamental sinus waveform signal with a harmonic series was simulated. The results are almost the same as the resampling of the fundamental sinus waveform signal with a single swept harmonic. The harmonic series is made up of an odd number order of the harmonic signals. All the harmonic signals were set up with a 10% amplitude value of the fundamental sinus waveform signal. The influence of the harmonic interactions on the fundamental sinus waveform signal or on each harmonic signal is slightly higher than the resampling with the single harmonic.

**Figure 9:**
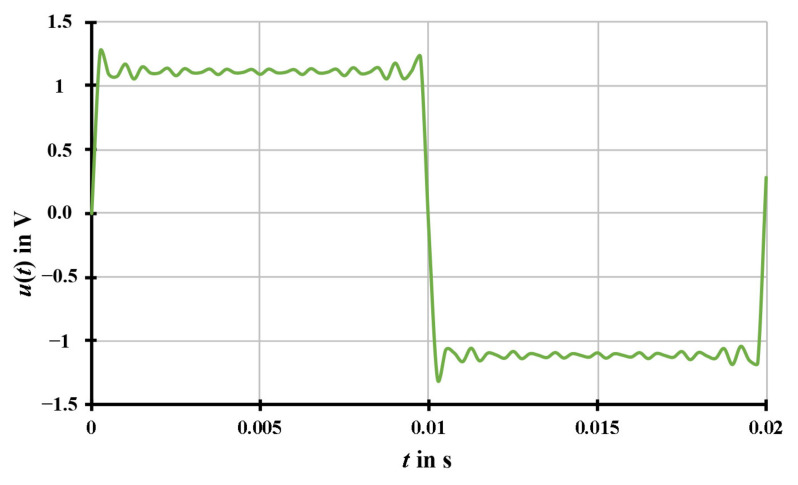
The generated square waveform at 50.1 Hz based on the harmonic series. The signals are simulated with parameters of *f*_S_ = 4000 Hz, *T*_W_ = 1 s, *U*_1_ = 1 V, *f*_1_ = 50.1 Hz, *φ*_1_ = 0°, *U*_H_ = 1/(2*h* + 1)*·U*_1_, *f*_H_ = (2*h* + 1)*·f*_1_, and *φ*_H_ = 0°, where *h* is an integer and *h* = 0, 1, 2, …, 15, without noise signal.

Finally, the resampling of a square waveform, shown in Figure 9, was simulated based on the harmonic series. The order of the simulated harmonic signal is 2*h* + 1, where *h* is the positive integer number, whereby a maximum harmonic number of 31 has been used in the simulation. The amplitude of the simulated harmonic signal decreases with 1/(2*h* + 1) of the amplitude of the fundamental signal.

**Figure 10:**
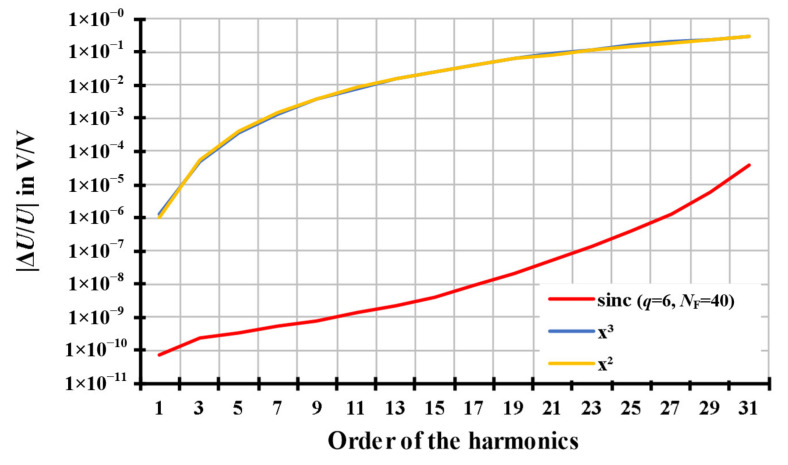

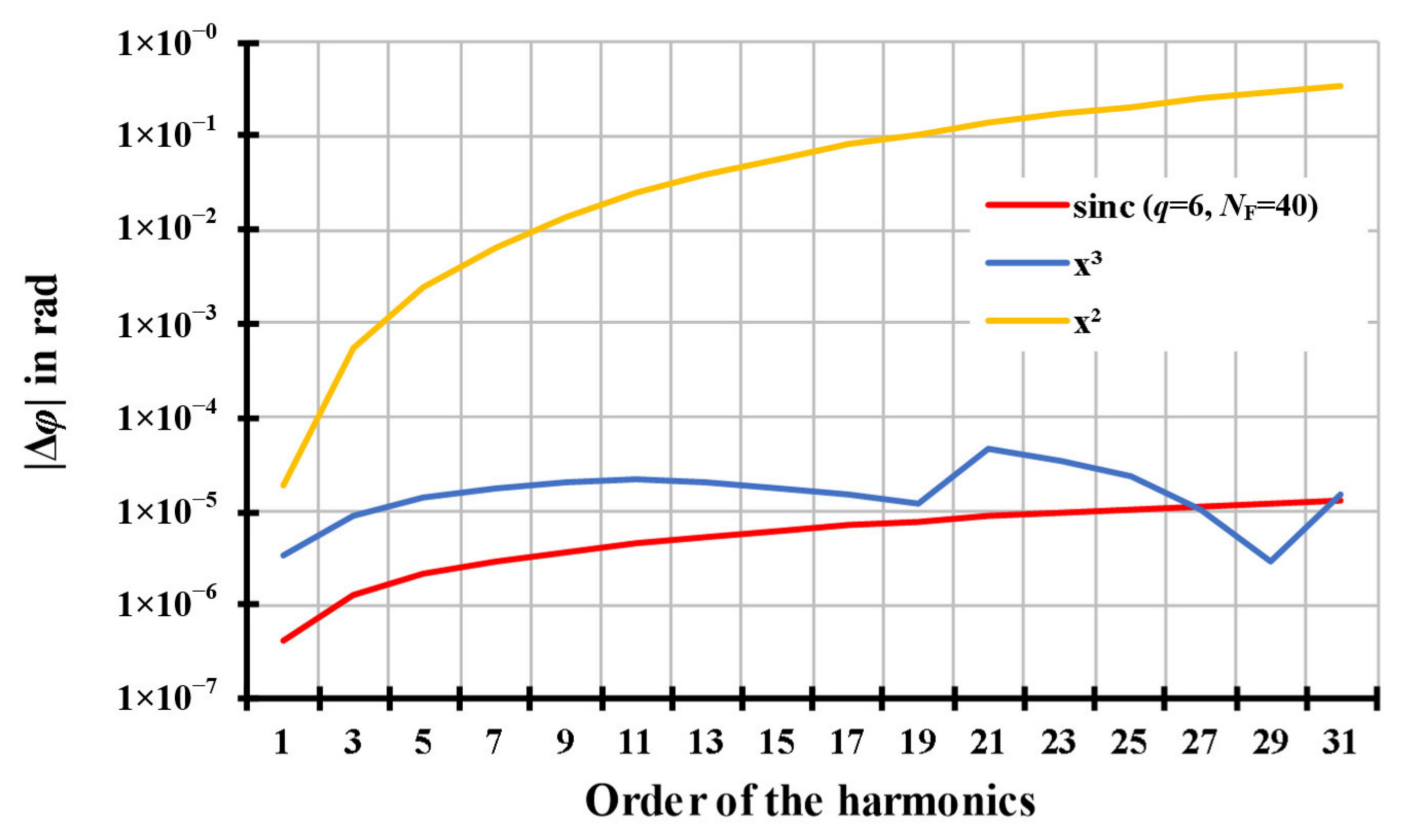
The resampling amplitude and phase error of a square waveform signal. The signals are simulated with parameters of *f*_S_ = 4000 Hz, *T*_W_ = 1 s, *U*_1_ = 1 V, *f*_1_ = 50.1 Hz, *φ*_1_ = 0°, *U*_H_ = 1/(2*h* + 1)*·U*_1_, *f*_H_ = (2*h* + 1)*·f*_1_, and *φ*_H_ = 0°, where *h* is an integer and *h* = 0, 1, 2, …, 15, without noise signal. The orange curves represent the results of the interpolation based on the quadratic function. The blue curves represent the results of the interpolation based on the cubic function. The red curves represent the results of the modified.

The corresponding resampling amplitude and phase error of the square waveform signal are displayed in Figure 10.

For the relative amplitude errors, the results of the resampling algorithms based on the quadratic and the cubic interpolations, shown as the orange curves and the blue curves, respectively, in Figure 10 (above), are practically identical. The relative amplitude errors of the square waveform signal range from 10^−6^ *V*/*V* to 0.2 *V*/*V*. The relative amplitude errors of the modified sinc algorithm, shown as the red curves in Figure 10 (above), are several orders more accurate than the quadratic and the cubic algorithms. The relative amplitude errors of the fundamental signal are below 10^−9^ *V*/*V* and below 10^−4^ *V*/*V* for the 31st harmonic of square waveform signal.

For the absolute phase errors, except quadratic interpolation, the results of the resampling algorithms based on the cubic function and the modified sinc interpolation, shown as the blue curves and the red curves, respectively, in Figure 10 (below), are below 10^−4^ rad at all harmonics with their order from 3 to 31.

### 3.2. Experimental Results Based on the Microcontroller-Based SV Devices

**Figure 11:**
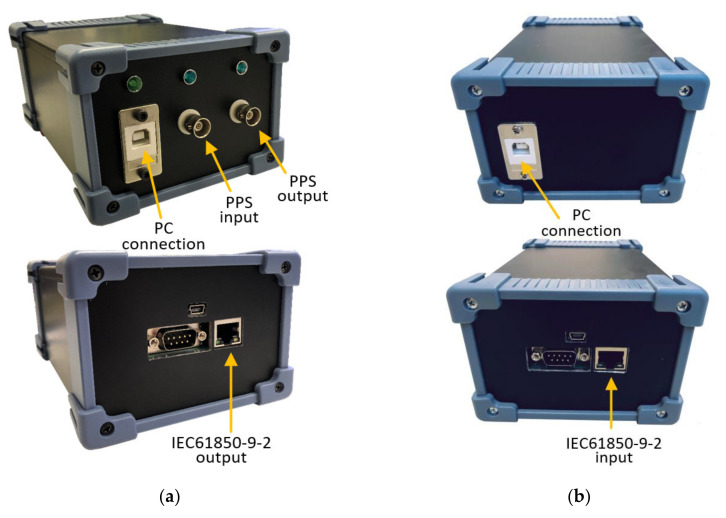
(**a**) Photo of the microcontroller-based SV generator box with additional port information. (**b**) Photo of the microcontroller-based SV receiver box with additional port information.

The laboratory experiments were executed according to the emulation of the SV-based communication, shown in Figure 5 in Section 2.2. The developed microcontroller-based SV generator box (shown in Figure 11a) and SV receiver box (shown in Figure 11b) have been developed for the laboratory experiments.

The developed SV generator box (shown in Figure 11a) is a device that sends pre-programmed SV data over ethernet using the IEC 61850-9-2 protocol. The basic module is a 32-bit ARM Cortex-M4 CPU with ethernet and USB port. The SV data can be programmed with a PC using the USB connection. The SV generator box can send three-phase four-wire current and voltage signals at the same time. The programmed current and voltage values and the phase angle can be differently set by the user. The sampling frequencies *f*_S_ can be set to 4 kS/s, 4.8 kS/s, 12.8 kS/s or 14.4 kS/s according to the standard IEC 61869-9. The developed SV receiver box (shown in Figure 11b) is a device that receives the SV data over ethernet using the IEC 61850-9-2 protocol. The basic module is a 32-bit ARM Cortex-M4 CPU with ethernet and USB port. The SV receiver box gathers all the SV data and provides PC with a time buffer for the SV data processing. The SV data is sent to a PC using the USB connection

According to the simulation results in Section 3.1, the experimental measurements with the developed SV devices are using the modified sinc algorithm for the resampling process. The measurements were processed with three different voltages of *U*_1_ = 1 V/100 V/100 kV for comparison. The results are firstly saved from the LabView program as the data set, then analysed and presented in the diagrams. As a result, the experimental measurement based on SV device results are presented as: (i). the difference between the generated signals, set by the PC and the quantized signals, sent by SV generator box, (ii). the spectra composition of the received signals by the SV receiver box and of the resampled signals after applying the modified sinc algorithm.

**Figure 12:**
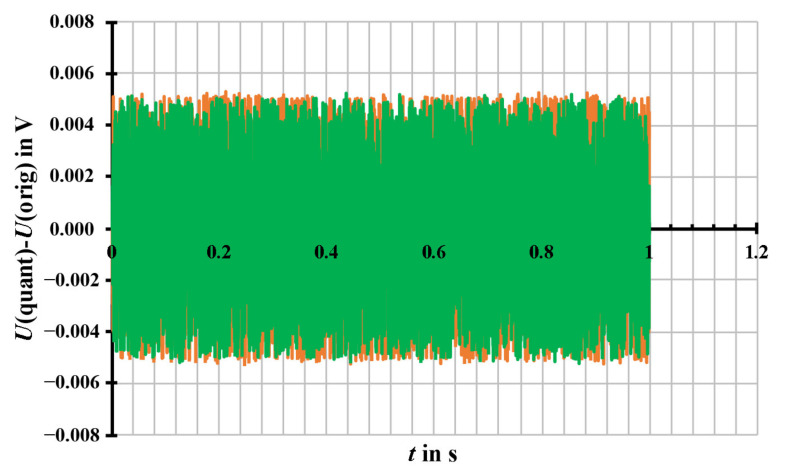
The difference between the generated signals, set by the PC and the quantized signals, sent by SV generator box (green: *U*_1_ = 1 V, orange: *U*_1_ = 100 V) in time domain. The signals are simulated with parameters of *f*_S_ = 4000 Hz, *T*_W_ = 1 s, *f*_1_ = 50.1 Hz, and *φ*_1_ = 0 ° with different RMS values of the amplitude *U*_1_ = 1 V/100 V, without noise and harmonics.

**Figure 13:**
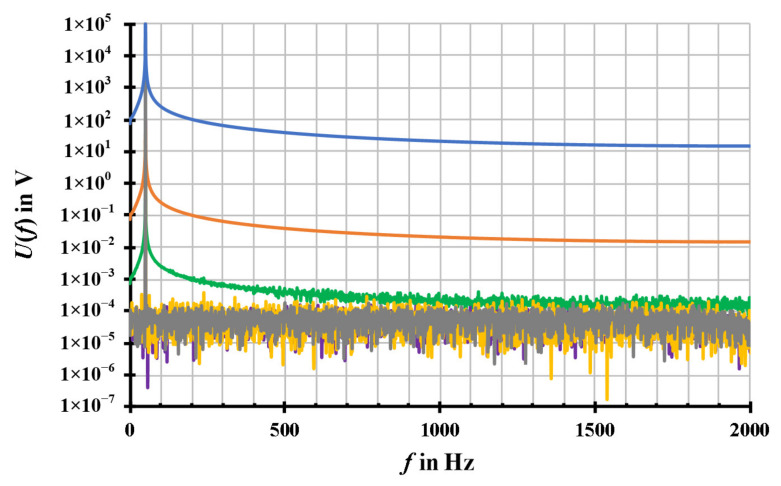
Full DFT spectra of three received signals from SV receiver box without resampling processing (green: *U*_1_ = 1 V, orange: *U*_1_ = 100 V, blue: *U*_1_ = 100 kV) and the three corresponding resampled signals by using the modified sinc algorithm (yellow *U*_1_ = 1 V, purple: *U*_1_ = 100 V, grey: *U*_1_ = 100 kV). The signals are simulated with parameters of *f*_S_ = 4000 Hz, *T*_W_ = 1 s, *f*_1_ = 50.1 Hz, and *φ*_1_ = 0° with different RMS values of the amplitude *U*_1_ = 1 V/100 V/100 kV, without the noise and the harmonic series. The modified sinc algorithm are simulated with parameters of *q* = 6 and *N*_F_ = 40.

According to Figure 12, for instance, the results of the signals with *U*_1_ = 1 V and *U*_1_ = 100 V are presented, which show the difference between the generated signals, set by the PC and the quantized signals, sent by SV generator box in time domain. As a consequence, the signals with various amplitude, *U*_1_ = 1 V/100 V/100 kV, have eventually the same rectangular error distribution of ± 5 mV. This quantisation noise, which is due to the prescribed limited number resolution of the SV communication protocol, is independent of the signal amplitude values. Furthermore, the programmed quantization of 10 mV of the SV generator box is valid for acting as a SAMU in the hardware loop. With the chosen sampling parameters of *f*_S_ = 4 kHz and the window *T*_W_ = 1s (frequency resolution *f*_res_ = 1/*T*_W_), the noise floor in the spectra agrees with the theoretical level of (*U*_Noise_/√3)/√(*f*_S_/2) · √(*f*_res_). For a noise level of ± 5 mV, the noise floor is about 60 µV and is evenly distributed across the frequency range up to *f*_S_/2 (shown in Figure 13).

The full DFT spectra of three received signals by the SV receiver box and the three corresponding resampled signals after applying the modified sinc algorithm are presented in Figure 13. From the comparison of the signals before and after resampling processing, it is obvious to see that the resampled signals by using the modified sinc algorithm (yellow, purple, grey) has no more leakage that exists in the received signals from SV receiver box without resampling processing (green, orange, blue). The noise of the resampled signals of the signals with different RMS values are almost identical by about 60 µV. These noise results in frequency domain (shown in Figure 13) confirm the noise results in time domain (shown in Figure 12). The modified sinc algorithm is valid for the practical situation. Additionally, based on the SV devices, the experimental RMS amplitude values and phase values of the three received signals without resampling processing, the experimental RMS amplitude values and phase values of the three resampled signals by using the modified sinc algorithm as well as the corresponding errors between the signals with and without resampling processing are presented in Table 2.

From Table 2, in the first place it is concluded that, even for the non-realistic case in high-voltage system of 1V, the relative amplitude errors of three different signals with the modified sinc resampling algorithm based on the SV devices are in the order of 10^−4^ *V*/*V* and the absolute phase errors are in the order of 10^−4^ rad. These results are caused by the data quantization of 10 mV of the SV generator box. Moreover, the error of the frequency determination (+ 1.16 µHz/Hz) is in agreement with the phase error of 180 µrad.

Moreover, the errors with the higher voltages are proportionally better than the errors with the lower voltages, since the 10 mV quantization of the SV generator box has much less influences on the high voltages (e.g., 100 kV) compared to the low voltage (e.g., 1 V). In practical circumstance, for primary voltages above 100 V and primary current above 10 A, the errors due to the quantization of the SV protocol are in the order of 10^−6^ *V*/*V* for the relative amplitude errors and in the order of 10^−6^ rad for the phase errors.

## 4. Conclusions

A modified sinc interpolation method with a finite impulse response (FIR) has been presented. The resampling based on the modified sinc algorithm takes the longest computation time, compared to the other two algorithms. The results of the modified sinc resampling algorithm are the most accurate within a normalised bandwidth of up to 40% of *f*_S_. Even with strongly distorted waveforms, the amplitude and phase errors of the modified sinc algorithm are well below 10 ppm and μrad with signals of up to 40% of *f*_S_. The deviation of the results for the RMS and phase angle is in the order of 10^−8^ *V*/*V* (or rad) for normalised frequencies of up to 20% of the sampling frequency. No practical degradation in the presence of noise and harmonics could be observed. 

To sum up, the results of the modified sinc interpolation method with the phase synchronisation were simulated for typical sampling settings of sampled value-based digital instrumentation in high voltage substations. As the simulation results showed, the proposed resampling process allows the synchronisation of the sampled values to PPS with almost mathematically perfect accuracy. In addition, the experimental results based on the microcontroller-based SV devices confirmed that the proposed modified sinc interpolation method works with SV-based instrumentation as well. The simulations and experimental results allow the highly accurate implementation of the proposed resampling process in the future SV-based calibration systems for SV-based instrumentation, such as digital instrument transformers, SAMUs and digital energy meters. As an example, the highest accuracy class of a SAMU is 0.05 [28]. Calibration systems for such a SAMU should be an order of magnitude more accurate. With the modified sinc interpolation method, no practical degradation of the measurement uncertainty through different sampling processes in the device under test (i.e., the SAMU) and the reference (i.e., the calibration system) is to be expected. As a consequence, the modified sinc interpolation method is currently adapted to a calibration platform in the laboratory for the current transformer measuring system. Based on the accurate processing of the digital signal quantities by using the modified sinc resampling algorithm, further power-quality-related calibrations will be accomplished in the future. 

## Figures and Tables

**Table 1:** The standard sample rates for the SV-based instruments according to the standard IEC 61869-9 [3]. The marked sample rates (*) are preferred for digital interface to instrument transformers.

Sample Rate in Hz	ASDU Per Frame	Publishing Rate Frame/s	Frequency in Hz
4000	1	4000	50
4800	1	4800	50/60
4800 *	2	2400	50/60
5760	1	5760	60
12,800	8	1600	50
14,400 *	6	2400	50/60
15,360	8	1920	60
96,000 *	1	96,000	wideband

**Table 2:** Experimental SV device-based amplitude and phase values of the three received signals without resampling processing and of the three resampled signals by using the modified sinc algorithm as well as the three corresponding errors between the signals with and without resampling processing. The signals are simulated with parameters of *f*_S_ = 4000 Hz, *T*_W_ = 1 s, *f*_1_ = 50.1 Hz, and *φ*_1_ = 0 ° with different RMS values of the amplitude *U*_1_ = 1 V/100 V/100 kV, without the noise and the harmonic series. The modified sinc algorithm are simulated with parameters of *q* = 6 and *N*_F_ = 40.

*U*_1_ in V	*Φ*_1_ In Rad	*f*_1_ in Hz	*U*_DFT_(*f*_1_) in V	*φ*_DFT_(*f*_1_) in Rad	(*U*_DFT_(*f*_1_) − *U*_1_)/*U*_1_ in %	*φ*_DFT_(*f*_1_) − *φ*_1_ in Crad
1	0	50.100058	9.998 × 10^−1^	1.77 × 10^−4^	−0.020	0.018
100	0	50.100001	1.000 × 10^2^	1.56 × 10^−6^	0.000	0.000
100,000	0	50.100000	1.000 × 10^5^	2.13 × 10^−9^	0.000	0.000

**Figure A1:**
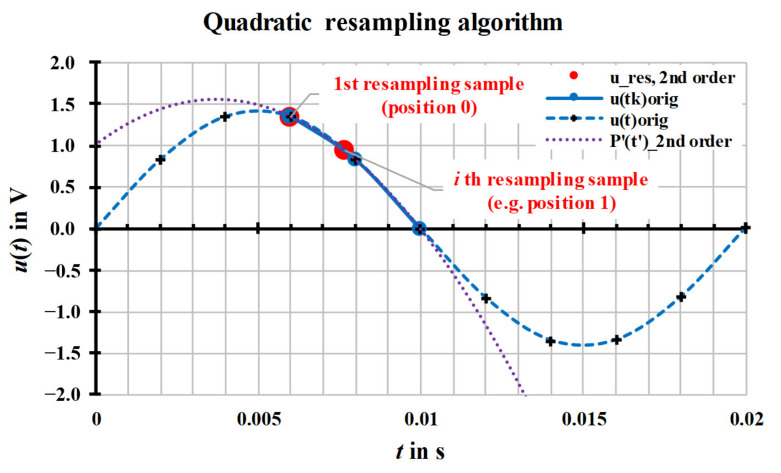
Detailed figure for calculating sample(s) based on a quadratic interpolation. The signals are simulated with parameters of *f*_S_ = 500 Hz, *T*_W_ = 1 s, *U*_1_ = 1 V, *f*_1_ = 50.1 Hz, *φ*_1_ = 0 grad, and *T*_Del_ = 0.005 s without any noise or any harmonics. The blue dashed curve with the black crosses represents the waveform of the sampling signal sequence. The three blue dots represent the selected sampling samples for the resampling process. The purple dotted curve represents the determined quadratic function. The red dots represent two resampling samples.

**Figure A2:**
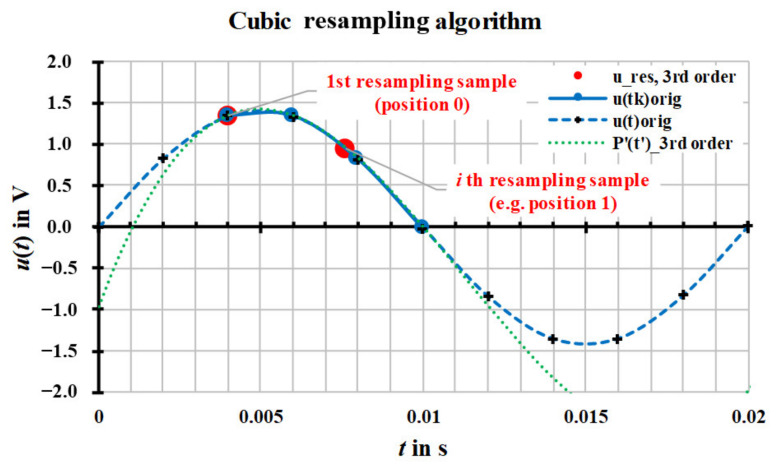
Detailed figure for calculating sample(s) based on a cubic interpolation. The signals are simulated with parameters of *f*_S_ = 500 Hz, *T*_W_ = 1 s, *U*_1_ = 1 V, *f*_1_ = 50.1 Hz, *φ*_1_ = 0°, and *T*_Del_ = 0.003 s without the noise and the harmonic series. The blue dashed curve with the black crosses represents the waveform of the sampling sequence. The four blue dots represent the selected original samples for the resampling process. The green dotted curve represents the determined cubic function. The red dots represent the samples after the resampling process.

## Appendix A

Appendix A supplements Section 2.1. The detailed operations of the resampling algorithms based on the quadratic interpolation and the cubic interpolation are presented.

### Appendix A.1. Quadratic Interpolation

Referring to the resampling algorithm of this work, the interpolation based on a quadratic function deals with the coefficient derivation of a quadratic function from three sampling samples of the given signal sequence *u*[*k*]. With the selection of the first sample for one resampling process, the following three samples are determined sequentially with its next two samples and are expressed as (*t*_0_, *u*[*t*_0_]), (*t*_1_, *u*[*t*_1_]), (*t*_2_, *u*[*t*_2_]). The initial quadratic function for the interpolation is assumed as follows:

*P*(*t*) = *a*_0_ + *a*_1_*t*+ *a*_2_*t*^2^. (A1)

The detailed resampling calculation of the interpolation based on a quadratic function is illustrated in Figure A1. The *i*th resampling process deals with three given sampling samples: one nearest sampling sample before the resampling sample and two nearest sampling samples after the resampling sample. The detailed determination of the first resampling sample (position 0 in Figure A1) is explained in Section 2.1.4.

The resampling process, for instance, the *i*th resampling sample shown in Figure A1, explains one of the *N*’ resampling processes under the circumstance with the given parameters. The process is divided into four parts. Firstly, the position of the resampling sample (position 1 in Figure A1) is determined by the index *i*. Secondly, the three sequential sampling samples (the three blue dots in Figure A1) are selected from the sampling signal sequence. The resampling sample should be, as designed, located between the first and second sampling samples. Thirdly, the quadratic function (the purple dotted curve in Figure A1) for the interpolation is acquired through the three sampling samples. Finally, the resampling value is calculated through the quadratic function.

For a simpler and faster calculation, the origin of the coordinate system is moved to the first original sampling sample and the x-axis is normalised to *T*_S_. The new quadratic function *P*’(*t*’) in the auxiliary coordinate system for the resampling process is defined as follows:

*P’*(*t*’) = *a*_0′_ + *a*_1′_*t*’+ *a*_2′_(*t*’)^2^, (A2)

where *t*’ is the normalised time (*t* − *k*·*T*_S_)/*T*_S_. With the three sequential original sampling samples in the auxiliary coordinate system (0, *u*(0)), (1, *u*(1)), (2, *u*(2)), the quadratic function is determined with the coefficient matrix $\vec{a^{\prime }}$:

(A3) $$\vec{a^{\prime }}=\left[\begin{array}{ccc} 1 & 0 & 0\\ -\frac{3}{2} & 2 & -\frac{1}{2}\\ \frac{1}{2} & -1 & \frac{1}{2} \end{array}\right]\cdot \vec{u},$$

where $\vec{u}=\left[\begin{array}{c} u\left(0\right)\\ u\left(1\right)\\ u\left(2\right) \end{array}\right]$.

### Appendix A.2. Cubic Interpolation

Referring to the resampling algorithm of this work, the interpolation based on a cubic function deals with the coefficient derivation of a cubic function from four sampling samples of the given signal sequence *u*[*k*], generated according to Equation (1). With the selection of the first sample for one resampling process, the following three samples are determined sequentially with the next three samples, expressed as (*t*_0_, *u*[*t*_0_]), (*t*_1_, *u*[*t*_1_]), (*t*_2_, *u*[*t*_2_]), (*t*_3_, *u*[*t*_3_]). The initial quadratic function for the interpolation is assumed as follows:

*P*(*t*) = *b*_0_ + *b*_1_*t*+ *b*_2_*t*^2^+ *b*_3_*t*^3^. (A4)

The detailed resamples calculation of the interpolation based on a cubic function is illustrated in Figure A2. The *i*th resampling process deals with four given sampling samples: the two nearest sampling samples before the resampling sample and the two nearest sampling samples after the resampling sample. The detailed determination of the first resampling sample (position 0 in Figure A2) is explained in Section 2.1.4.

Similar to the quadratic resampling process, the *i*th resampling sample shown in Figure A2, explains one of the *N*’ resampling processes under the circumstance with the given parameters based on cubic interpolation. The process is divided into four parts. Firstly, the position of the resampling sample (position 1 in Figure A2) is determined by the index *i*. Secondly, the four sequential sampling samples (the four blue dots in Figure A2) are selected from the sampling signal sequence. The resampling values should be, as designed, located between the second and third sampling samples. Thirdly, the cubic function (the purple dotted curve in Figure A2) for the interpolation is acquired through the four sampling samples. Finally, the resampling value is calculated through the cubic function. To allow simpler and faster calculations, the origin of the coordinate system is moved to the first original sample and the x-axis is normalised to *T*_S_. The new cubic function *P*’(*t*’) in the auxiliary coordinate system for the resampling process is defined as follows:

*P’*(*t*’) = *b*_0′_ + *b*_1′_*t*’+ *b*_2′_(*t*’)^2^+ *b*_3′_(*t*’)^3^, (A5)

where *t*’ is the normalised time (*t* − *k*·*T*_S_)/*T*_S_. With the four sequential samples in the auxiliary coordinate system (0, *u*(0)), (1, *u*(1)), (2, *u*(2), (3, *u*(3)), the cubic function is determined with the coefficient matrix $\vec{b^{\prime }}$:

(A6) $$\vec{b^{\prime }}=\left[\begin{array}{cc} \begin{array}{cc} 1 & 0\\ -\frac{11}{6} & 3 \end{array} & \begin{array}{cc} 0 & 0\\ -\frac{3}{2} & \frac{1}{3} \end{array}\\ \begin{array}{cc} 1 & -\frac{5}{2}\\ -\frac{1}{6} & \frac{1}{2} \end{array} & \begin{array}{cc} 2 & -\frac{1}{2}\\ -\frac{1}{2} & \frac{1}{6} \end{array} \end{array}\right]\cdot \vec{u},$$

where $\vec{u}=\left[\begin{array}{c} \begin{array}{c} u\left(0\right)\\ u\left(1\right) \end{array}\\ \begin{array}{c} u\left(2\right)\\ u\left(3\right) \end{array} \end{array}\right]$.
